# The Relationship between pH and Bacterial Communities in a Single Karst Ecosystem and Its Implication for Soil Acidification

**DOI:** 10.3389/fmicb.2016.01955

**Published:** 2016-12-16

**Authors:** Yuan Yun, Hongmei Wang, Baiying Man, Xing Xiang, Jianping Zhou, Xuan Qiu, Yong Duan, Annette S. Engel

**Affiliations:** ^1^Geomicrobiology Group, State Key Laboratory of Biogeology and Environmental Geology, China University of GeosciencesWuhan, China; ^2^Laboratory of Basin Hydrology and Wetland Eco-restoration, China University of GeosciencesWuhan, China; ^3^Department of Earth and Planetary Sciences, University of Tennessee, KnoxvilleTN, USA

**Keywords:** cave ecosystem, bacterial diversity, acidification, pH, overlying soil, Illumina sequencing

## Abstract

Enhanced monsoon duration and soil acidification from acid rain are expected to impact the distribution of microbial communities in surface and subsurface environments, although these impacts are poorly understood for most systems. In central China, soluble carbonate bedrock forms extensive karst landscapes. Current predictions are that the amount of monsoonal precipitation and acid rainfall in central China will increase, which is expected to lead to changes in the pH balance of karst ecosystems. To evaluate the role of pH, total organic carbon, and other geochemical parameters (e.g., Ca^2+^, Mg^2+^, NH_4_^+^, NO_x_, SO_4_^2-^) in shaping bacterial communities within a single karst system in central China, samples were collected from the thin surface soils overlying Heshang Cave, cave sediments, and weathered cave passage rocks from the entrance, twilight, and dark zones, as well as from epikarstic drip waters inside the cave. Illumina sequencing of 16S rRNA genes and multivariate statistical analyses revealed that each tested community was distinct and the community variability was significantly correlated with pH, total organic carbon, and potassium concentrations. Specifically, surface soils were dominated by Acidobacteria, Verrucomicrobia and Planctomycetes, and diversity significantly decreased with acidic pH values. Nitrospirae, Gemmatimonadetes, Firmicutes, and Chloroflexi were unique to cave sediments, while Actinobacteria and Proteobacteria dominated weathered rocks and drip waters, respectively. The results reveal important implications regarding the effects of acidification on bacterial communities in karst areas, and on the control of pH in shaping bacterial communities throughout a karst system. Increased water flux into and through karst habitats due to monsoonal precipitation may result in deeper penetration of acidic solutions into karst and shift the bacterial communities inside the cave in the future.

## Introduction

**FIGURE 1:**
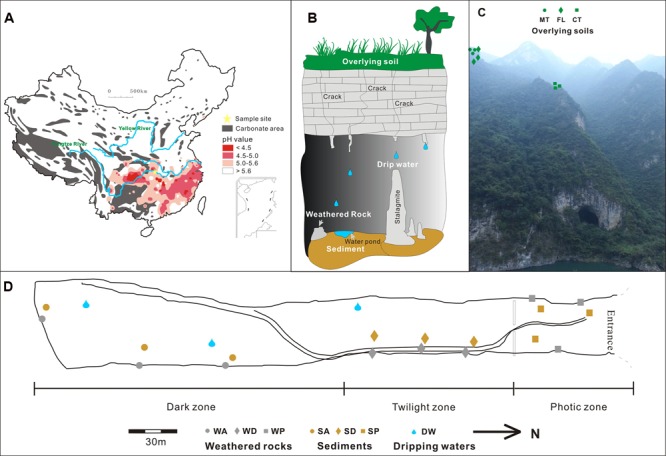
**Location of study site on the map of carbonate and acid rain distribution in China (A)** modified after http://english.mep.gov.cn/standards_reports/soe/soe2011/201307/t20130712_255427.htm and Yuan and Cao (2008) **(B)**. The schematic of the vertical section of Heshang Cave ecosystem, Hubei Province, central China, modified after Yang et al. (2011) and sampling sites outside and inside Heshang Cave are shown in **(C,D)**, respectively. Different colors denote samples of overlying soil (green), weathered rock (gray), sediment (brown), and dripping water (blue). MT, soils on the mountaintop; CT, soils above the cave; FL, soils from the nearby farm land; WA, weathered rocks in dark zone; WD, weathered rocks in twilight zone; WP, weathered rocks in photic zone; SA, sediments in dark zone; SD, sediments in twilight zone; SP, sediments in photic zone; DW, drip waters.

The 540,000 km^2^ karst region in eight provinces of central China (including Yunnan, Sichuan, Chongqing, Guizhou, Hunan, Hubei, Guangdong, and Guangxi provinces) (102–111° E, 23–32° N) is the key zone of east Asia karst areas, which is one of the three largest karst areas in the world (Fan et al., 2011). The central China karst is experiencing severe acid rain recently due to anthropogenic activities and increased precipitation from enhanced monsoon durations (**Figure 1A**). Widespread ecological and agricultural consequences are expected to include soil acidification (Larssen et al., 2006; Duan et al., 2011; Xu et al., 2015).

Karst landscapes typically have thin soils covering soluble carbonate bedrock (e.g., limestone or dolomite). At present, however, there is limited understanding of karst soil bacterial communities in central China. Previous research demonstrates that pH variations shape the dominant bacterial groups in different types of soils (Rousk et al., 2010; Bartram et al., 2014) and across different geographical scales (Fierer and Jackson, 2006; Baker et al., 2009; Jones et al., 2009; Shen et al., 2013; Liu et al., 2014), with soil pH correlating to the presence and relative abundances of specific taxonomic groups, such as Alpha-, Beta-, Gammaproteobacteria, Actinobacteria, and Acidobacteria subgroups 4–7 (Rousk et al., 2010; Shen et al., 2013). It is expected that as precipitation increases in central China, especially acid rainfall, then soil pH will decrease and subsequently affect microbial communities (Pu et al., 2011). Indeed soil acidification in other parts of China has already been linked to lower microbial abundances in soils (Wu et al., 2006; Xu et al., 2015), as well as diminished microbial diversity (Zhalnina et al., 2015).

In karst systems, as acidic meteoric water percolates through the carbonate rocks in the unsaturated epikarst zone, more rock dissolution should occur at shallower depths than if the meteoric water was of neutral pH (Ford and Williams, 2007). Changes in epikarst solution chemistry, and the depths to which acidic epikarst solutions become buffered by carbonate rock dissolution, would impact how fast water can move through the epikarst, as well as the depth to which biogeochemical processes occur within the epikarst (Ford and Williams, 2007). These chemical changes would be expected to influence bacterial community growth and diversity within the karst system.

Therefore, we hypothesize that the compositional variability among bacterial communities in the different karst habitats would reflect spatial pH changes, such that communities from surface karst habitats (i.e., overlying soils) would strongly correlate to pH, but those from deeper karst habitats (e.g., drip water, cave sediments, weathered rock) would not be significantly affected by pH change due to greater pH buffering capacity of the habitat. To test the hypothesis, we investigated the correlation between environmental factors, especially pH, and microbial communities in a full karst ecosystem, from the surface soils overlying the cave system and from various habitats within the cave, including epikarstic drip waters that link the surface to subsurface (**Figure 1B**). Although microbial communities have been separately investigated from drip waters in caves (Liu et al., 2010; Yun et al., 2016), cave sediments (Man et al., 2015; Wu et al., 2015), weathered rocks in caves (Ward et al., 2009; Man et al., 2015), soils overlying cave systems (Castro et al., 2010; Ortiz et al., 2014), and bat guano (Man et al., 2015), no systematic evaluation of the communities throughout a karst system has been conducted previously. The results from this study provide an assessment of bacterial communities in central China karst and reveal the potential consequence of acidification on bacterial community composition in the karst habitats.

## Materials and Methods

### Site Description and Sampling

Heshang Cave (29°40′–30°48′ N, 108°30′–111°20′ E) is located in Changyang County, Hubei Province, China. The annual average temperature is ∼16.5°C and average annual rainfall is ∼1118 mm. Most of the precipitation (∼70%) occurs in April to September (Hu et al., 2008a; Yun et al., 2016). Vegetation consists of dense forests of subtropical tress and shrub, and corn and other vegetable crops are grown on the mountains. The cave is 250 m long and situated ∼30 m above Qingjiang River, a tributary to the Yangtze River, with a sole entrance ∼20 m in diameter (**Figure 1C**). The cave formed in Cambrian-aged dolomite (∼400 m thick), which is covered with a 20–40 cm thick soil (Hu et al., 2008b).

Three types of surface soils (sampling depth < 2 cm) were collected by five-point sampling method: four soil samples from the forested mountaintop (MT1/MT2/MT3/MT4), three soil samples from agricultural land (FL1/FL2/FL3) and three soil samples directly above the cave (CT1/CT2/CT3) (**Figure 1C**). Inside the cave, nine surface sediment samples (sampling depth < 1 cm, silt deposits) were collected from the photic zone near the cave entrance, twilight zone (i.e., the transition between the entrance and complete darkness), and the dark zone (i.e., complete darkness), with triplicate samples from each zone (**Figure 1D**). Nine soft weathered carbonate rock samples from the cave wall were also collected in each zone of the subterranean cave. Three drip water samples (DW1/DW2/DW3) were collected. DW3 was in the twilight zone, and DW1 and DW2 were in the dark zone (**Figure 1D**). Drip water samples were collected with 10 L sterile plastic bottles and sterile funnels, and the others were collected aseptically with 50 ml sterile plastic centrifuge tubes (Corning), as described previously (Yun et al., 2016). All samples were transported to the geomicrobiology laboratory in China University of Geosciences (Wuhan) under refrigeration within 24 h of collection. An aliquot of 200 ml drip water samples and a subsample of all the other samples were stored at 4°C for physicochemical property analysis. Sterile membrane filters (0.22 μm, 47 mm, Supor-200, Pall Corporation, USA) were used to filter from 3 to 10 L of the remaining water samples. The filters and remainder of other materials were stored at -80°C until DNA extraction.

### Physicochemical Analysis

pH of drip water was measured *in situ* with a multiparameter water quality detector (HACH, Loveland, CO, USA) (Yun et al., 2016). Solid samples were pre-frozen at -80°C for an hour and then freeze-dried (ALPHA 1-2 LD, Christ, Germany) for 48 h to remove water. An aliquot of 1 g freeze-dried solid sample was mixed with 5 ml distilled water. After shaking with an end-to-end shaker for 5 min, the mixture was centrifuged at the speed of 6,800 × *g* for 10 min. The supernatant pH was measured with a UB-7 pH meter (Denver Instrument) after calibrations to pH 4, 7, and 10.

Total organic carbon (TOC) content from the solid samples was analyzed with a C-S analyzer (EA 4000, Analytik Jena AG, Jena, Germany) with a 3 μg g^-1^ detection limit, using high temperature ceramic technology, and standard samples AR4007 (ALPHA, USA) and AR1034 (ALPHA, USA) were used to monitor quality. TOC of drip water samples was analyzed by a TOC analyzer (Vario TOC cube, Elementar, Hanau, Germany) with a detection limit of 6 ng g^-1^, using the high temperature catalytic oxidation method.

The concentrations of dissolved major anions and cations for the drip waters were measured from filtered samples. For solid samples, 2 g aliquots of freeze-dried material were mixed with 2 ml deionized water (1:1 w/v ratio) and then shaken for 10 min. After centrifugation at 2500 × *g* for 2 min supernatants were filtered through 0.22 μm membrane filters. The filtrates were analyzed using an ICS-600 ion chromatograph (Benedicto et al., 2014).

### DNA Extraction and 16S rRNA Gene Sequencing

Total nucleic acids were extracted from 0.5 g solid sample (dry weight) using the PowerSoil DNA Kit (MoBio Laboratories, Inc., USA) following the manufacturer’s instructions. For drip water samples, the PowerWater DNA Kit (MoBio Laboratories, Inc., USA) was used to extract DNA from the membrane filters (0.22 μm, 47 mm, Supor-200, Pall Corporation, USA) according to the manufacturer’s instructions. Bacterial diversity was examined after 250-bp paired-end amplicon sequencing using the primers 520F (5′-AYTGGGYDTAAAGNG-3′) and 802R (5′-TACNVGGGTATCTAATCC-3′) (Claesson et al., 2009) on an Illumina MiSeq platform in two separate runs at Shanghai Personal Biotechnology, Co., Ltd, (Shanghai, China).

Raw sequence reads were deposited in the NCBI Sequence Read Archive under the accession number PRJNA307221^1^.

### Data Analysis

Raw sequence data were quality filtered and analyzed using QIIME v 1.7.0 (Caporaso et al., 2010). Reads were processed by removing tags and primers, and the reads with an average quality score < 20 and read lengths < 150 bp were discarded. After being processed, reads were assembled by FLASH software^2^ with the overlap between R1 and R2 reads ≥ 10 bp. Combined with mothur software v 1.31.2 (Schloss et al., 2009), chimeric sequences were identified and removed using the method of UCHIME (Edgar et al., 2011). High-quality representative sequences for each operational taxonomic units (OTUs) were assigned using UCLUST (Edgar et al., 2011) with 97% sequence identity. Taxonomic classification was carried out using Greengenes 16S rRNA database 13_8 release^3^ with assignment tool of Blast^4^. Sample size for each sample was rarefied prior to diversity indices calculations, including the Simpson index^5^ and Shannon index^6^, and calculations were done with mothur software by using the command of ‘summary.single.’

A heatmap of relative abundance of bacterial phyla and OTUs distribution (with relative abundance > 2%) in each sample was performed with R software (v 3.2.0)^7^. A one-way ANOVA (analysis of variance) was calculated in SPSS (version 17.0)^8^ with the significant level of 0.05 to examine significance differences among the Simpson and Shannon index values. Significant taxonomic differences between the four different habitats (e.g., overlying soils, cave sediments, weathered rocks, and drip waters) were analyzed using the least discriminant analysis (LDA) effect size (Segata et al., 2011). This method was based on the factorial Kruskal–Wallis test (α = 0.05) among classes and the pairwise Wilcoxon test (α = 0.05) between subclasses, with one-against-all strategy for multi-class analysis, to identify taxa with significant differential abundances between categories. Significant taxa were used to illustrate the difference between each sample (Bokulich et al., 2014). To demonstrate the relationship between different samples, principal coordinate analysis (PCoA) was calculated based on weighted Unifrac results in QIIME (Caporaso et al., 2010). Redundancy analysis (RDA) was performed using Canoco 5.0 to reveal the effect of environmental factors on bacterial communities, and the significant factors were chosen according to *p*-value (<0.05, ter Braak and Smilauer, 2002). Regression analysis and analysis of variance were used to identify the correlation between the pH and Shannon index of each sample (Anderson-Cook, 2004) in SPSS (version 17.0) software.

## Results

### Geochemistry

**Table 1** summarizes the geochemical results. All solid and liquid samples were slightly alkaline, with pH ranging from 7.28 to 8.35, except four acidic mountaintop soils (**Table 1**). Compared with the drip waters that had low TOC values (<0.05%), TOC content was higher in overlying soils from the mountaintop and above the cave, as well as from weathered rock samples from the photic zone. NH_4_^+^ was not detected in drip water or in MT and FL soils, but was detected in CT soils. NO_3_^-^ and NO_2_^-^ content varied in soils, with NO_3_^-^ generally being higher than NO_2_^-^ in most soils; except in CT soils where NO_2_^-^ was higher than NO_3_^-^. Sulfate content was similar for all soil samples, ranging from 0.11 to 0.57 mM. Cave sediments and weathered rocks were chemically heterogeneous. Drip waters had higher NO_3_^-^ and SO_4_^2-^ concentrations compared with most of the soils, except the SO_4_^2-^ concentration in MT4, FL2, and CT3 (**Table 1**).

**Table 1:** Geochemical analysis of different samples from Heshang Cave ecosystem, central China.

Sample ID		pH	TOC (%)	Ca^2+^ (mM)	Mg^2+^ (mM)	K^+^ (mM)	Na^+^ (mM)	NH_4_^+^ (mM)	Cl^-^ (mM)	NO_2_^-^ (mM)	NO_3_^-^ (mM)	SO_4_^2-^ (mM)
Overlying soils	MT1	5.69	1.28	0.22	0.20	0.02	0.08	/	0.20	0.01	0.05	0.19
	MT2	5.40	1.29	0.44	0.30	0.03	0.15	/	0.63	0.01	0.06	0.21
	MT3	5.67	1.29	0.31	0.26	0.08	0.08	/	0.19	0.01	0.06	0.25
	MT4	4.05	1.32	0.14	0.18	0.18	0.08	/	0.20	0.01	0.07	0.57
	FL1	7.80	0.93	0.35	0.09	0.07	0.12	/	0.25	0.02	0.05	0.11
	FL2	7.64	0.64	0.52	0.16	0.34	0.09	/	0.18	0.05	0.09	0.44
	FL3	7.44	1.34	0.58	0.14	0.10	0.03	/	0.10	0.08	0.07	0.20
	CT1	7.70	2.50	1.58	0.66	0.08	0.05	0.59	0.36	0.21	0.03	0.20
	CT2	7.56	4.85	1.79	0.46	0.17	0.02	0.43	0.15	0.27	0.03	0.17
	CT3	7.28	4.98	2.26	1.50	0.13	0.13	0.26	0.54	0.12	0.05	0.42
Sediments	SA1	8.00	0.53	0.79	2.00	0.20	0.19	0.03	0.18	0.15	1.35	0.13
	SA2	8.20	0.56	1.89	3.56	0.14	0.19	0.02	0.20	0.11	11.28	0.19
	SA3	8.04	0.32	1.15	2.00	0.10	0.78	/	2.78	0.15	1.80	0.21
	SD1	8.35	0.67	0.72	1.09	0.12	0.08	0.19	0.15	0.26	0.23	0.18
	SD2	8.34	0.61	3.30	11.06	0.44	2.02	0.29	6.09	2.33	11.90	4.33
	SD3	7.75	0.16	6.58	11.71	0.31	0.79	0.03	2.20	0.08	/	2.71
	SP1	8.06	0.16	0.75	0.50	0.09	0.10	/	0.13	0.11	0.38	0.31
	SP2	8.20	0.25	0.69	0.43	0.08	0.09	/	0.15	0.07	0.39	0.40
	SP3	7.90	0.24	2.08	1.27	0.31	0.35	/	0.72	0.06	5.27	0.40
Weathered rocks	WA1	7.40	0.90	/	/	/	/	/	1.13	0.20	/	23.81
	WA2	7.90	0.32	0.67	/	/	/	0.12	0.14	0.13	1.07	0.30
	WA3	8.10	0.50	14.23	/	/	/	1.17	1.16	0.21	23.93	4.65
	WD1	7.99	0.36	0.78	1.53	0.59	0.19	0.07	0.33	0.12	1.73	0.44
	WD2	7.69	0.50	5.27	5.44	1.09	1.79	0.09	4.20	0.09	11.14	5.41
	WD3	7.84	0.23	0.98	0.98	0.69	0.35	0.22	0.83	0.10	0.94	0.26
	WP1	7.56	1.87	3.36	2.92	2.33	0.22	1.16	0.42	5.42	0.51	3.89
	WP2	7.86	2.02	/	10.37	2.39	1.01	0.34	1.99	2.87	0.22	2.14
	WP3	7.80	2.27	13.45	59.47	1.81	0.93	0.82	2.53	11.08	18.41	53.29
Dripping waters	DW1	7.86	0.01	0.89	1.50	0.02	0.13	/	0.08	0.18	0.14	0.33
	DW2	7.96	0.05	0.87	1.52	0.02	0.06	/	0.06	0.20	0.22	0.28
	DW3	7.76	0.01	0.68	1.85	0.02	0.06	/	0.05	0.20	0.20	0.33

*MT: soils on the mountaintop; CT: soils above the cave; FL: soils from the nearby farm land; WA: weathered rocks in dark zone; WD: weathered rocks in twilight zone; WP: weathered rocks in photic zone (i.e., entrance area); SA: sediments in dark zone; SD: sediments in twilight zone; SP: sediments in photic zone; DW: drip water.*

*/: below the detection limit.*

### Bacterial Community Compositions in Karst Habitats

**FIGURE 2:**
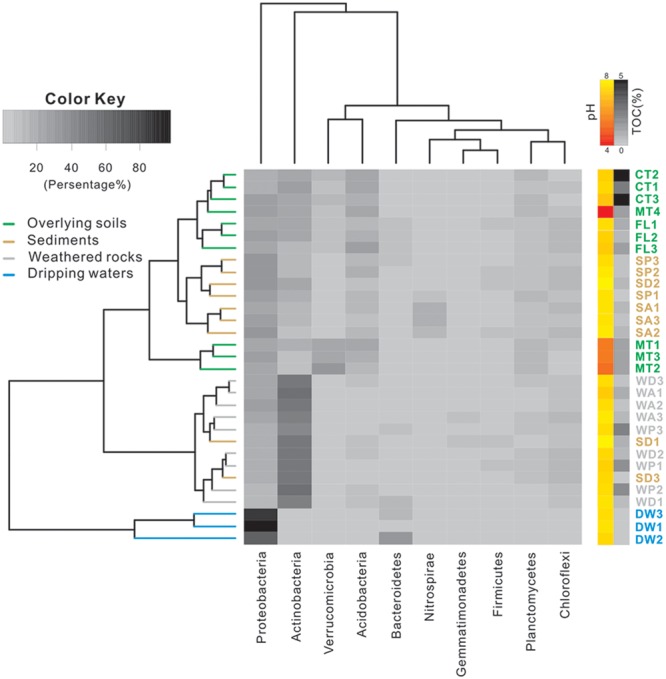
**Heatmap of 10 most abundant phyla in samples collected from Heshang Cave.** Different colors denote samples of overlying soil (green), weathered rock (gray), sediment (brown), and drip water (blue). The pH and TOC for each sample are shown on the right, with pH, from acidic (red), neutral (orange), to alkaline (yellow) conditions, and TOC content from low (gray) to high (dark) values. Abbreviations are the same as listed for **Figure 1**.

**FIGURE 3:**
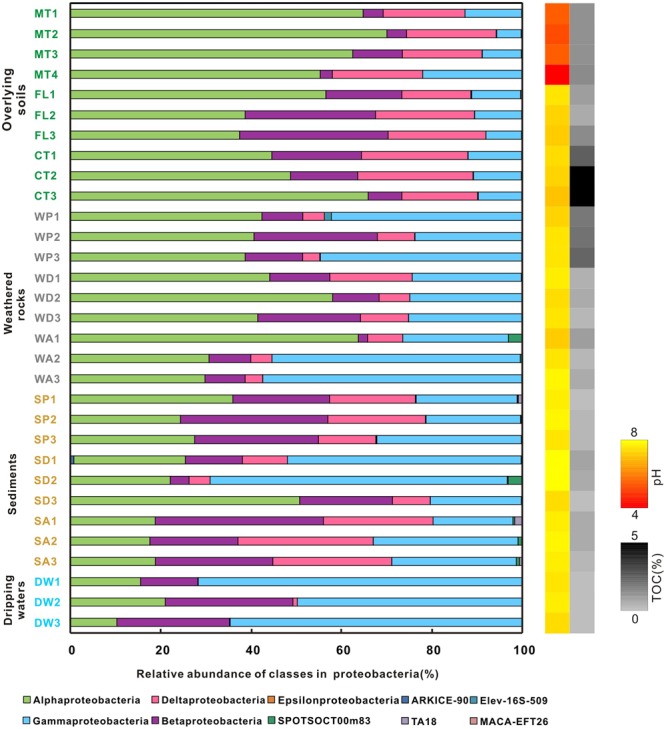
**Relative abundance of proteobacterial class from different habitats at Heshang Cave.** Different colors denote samples of overlying soil (green), weathered rock (gray), sediment (brown), and dripping water (blue). The pH and TOC for each sample are shown on the right, using a similar scheme as **Figure 2**. Abbreviations are the same as described for **Figure 1**.

At the phylum level, Proteobacteria and Actinobacteria were the most abundant groups in nearly all samples. For example, Proteobacteria comprised 20–40% of bacterial populations in some weathered rock samples (WA1, WA2, WA3, and WD3) and acidic surface soils (MT1, MT2, and MT3) and >60% in drip waters. Verrucomicrobia and Acidobacteria were also abundant in soils (**Figure 2**). Actinobacteria, Proteobacteria, and Acidobacteria were common in alkaline soils. Actinobacteria also dominated (40–60%) in all weathered rock samples and two sediment samples (SD1 and SD3). Within the Proteobacteria, Alphaproteobacteria were the most abundant in the acidic surface soils and some weathered rock samples with high TOC content (**Figure 3**), but their abundances decreased in the alkaline sediments and drip waters, which had the lowest TOC. Gammaproteobacteria and Betaproteobacteria were common in these low TOC samples (**Figure 3**).

**FIGURE 4:**
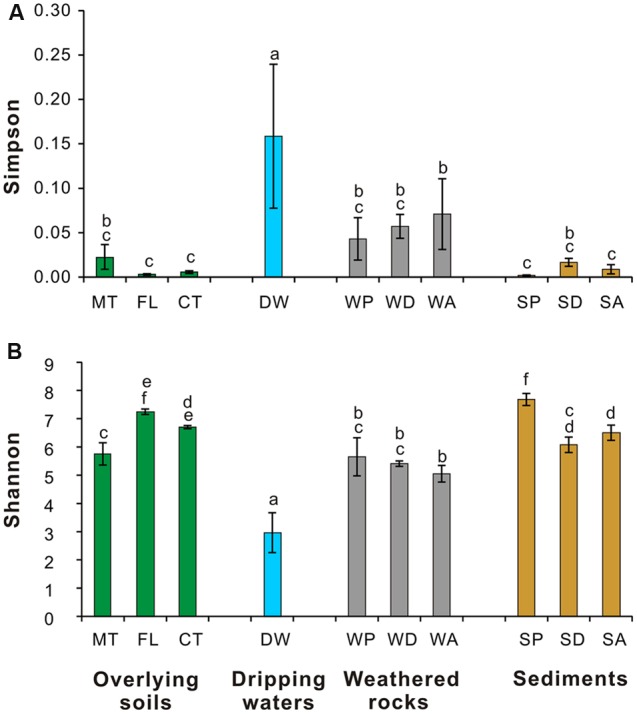
**Simpson **(A)** and Shannon **(B)** indices of 16S rRNA genes from microbial communities in different habitats at Heshang Cave.** See methods for description of the one-way ANOVA (analysis of variance) calculations. Different letters (a–f) above the bars showed significantly difference (*P* < 0.05) among each habitat according to Duncan’s multiple range test. Sample colors are the same as those in previous figures, and abbreviations described in **Figure 1**.

**FIGURE 5:**
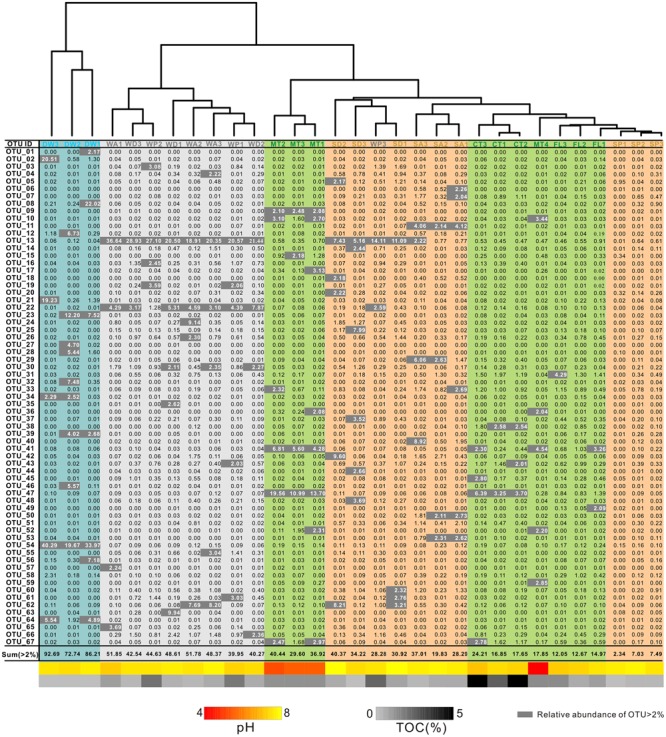
**Heatmap of operational taxonomic units (OTUs) (97% similarity) with relative abundance ≥ 2% from different habitats in the Heshang Cave ecosystem.** OTU compositions of overlying soils, weathered rocks, sediments, and drip waters are highlighted in green, gray, brown, and blue, respectively. The pH and TOC for each sample are shown at the bottom, with color schemes matching **Figure 2** and abbreviations described in **Figure 1**.

**FIGURE 6:**
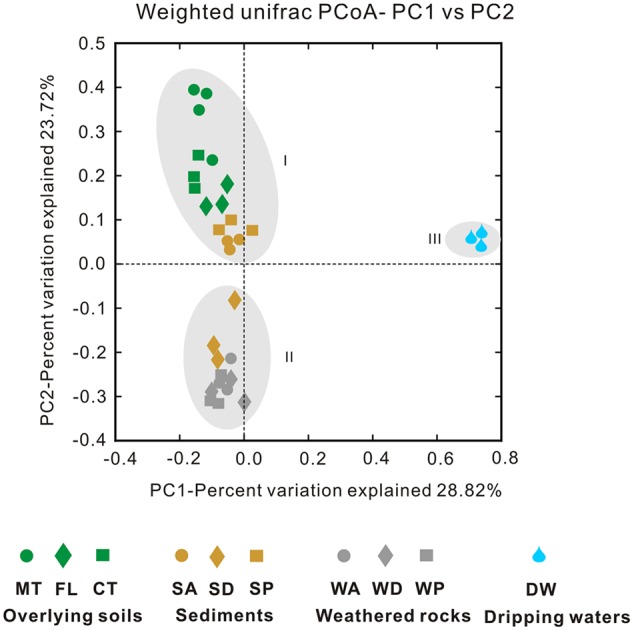
**Principal coordinate analysis (PCoA) plot based on weighted Unifrac results among samples collected from the Heshang Cave ecosystem.** Samples abbreviations are the same as previous figures, and abbreviations are described in **Figure 1**.

The Simpson (**Figure 4A**) and Shannon indices (**Figure 4B**) revealed that surface soils and cave sediments had the highest bacterial diversity and drip water samples had the lowest diversity. A cluster analysis of OTU-level diversity and PCoA based on weighted Unifrac results all revealed three distinct groups (**Figures 5** and **6**). Group I contained bacterial communities in all the overlying soils (MT, FL, and CT) and cave sediments in photic and dark zones. Group II consisted of bacterial communities from weathered cave rocks and twilight zone sediment samples. Group III was comprised of all drip water samples, and plotted at some distance away from the other groups in PCoA space (**Figure 6**).

**FIGURE 7:**
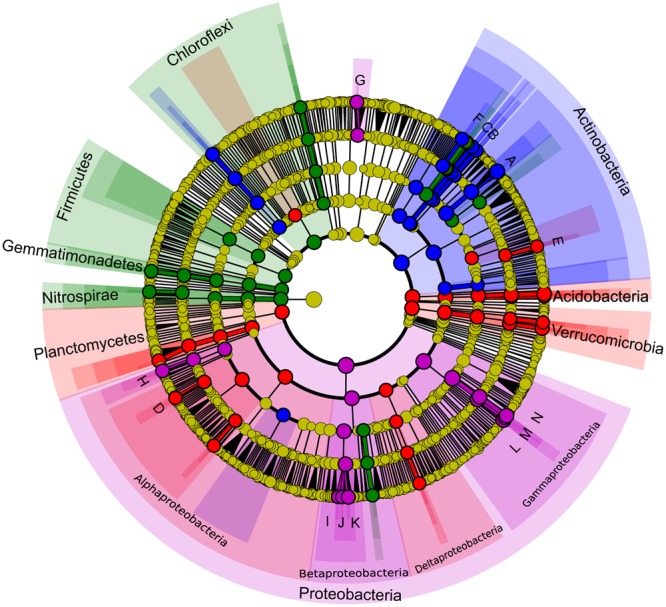
**Least discriminant analysis (LDA) effect size taxonomic cladogram comparing bacterial communities with a score higher than four.** Nodes from inside to outside circles represent the bacterial taxon from phylum to genus level, respectively. Significant discriminant taxon to a specific habitat and its branch areas are highlighted with red, green, blue and pink, which correspond to overlying soils, sediments, weathered rocks and drip water, respectively. Yellow nodes represent taxa that do not significantly discriminate between habitats. The diameter of the node is positively correlated with the relative abundance of the taxon. Abbreviations of discriminant genus are indicated: A, *Crossiella*; B, *Euzebya*; C, *Rubrobacter*; D, *Bradyrhizobium*; E, *Acidothermus*; F, *Gaiella*; G, *Sediminibacterium*; H, *Brevundimonas*; I, *Acidovorax*; J, *Hydrogenophaga*; K, *Polaromonas*; L, *Acinetobacter*; M, *Perlucidibaca*; N, *Pseudomonas*.

Least discriminant analysis effect size confirmed that each karst habitat had its own indicator taxa, from phylum to genus levels (**Figure 7**). Actinobacteria were specific to weathered rocks. Acidobacteria (18.85%), Verrucomicrobia (12.9%), and Planctomycetes (9.9%), Alphaproteobacteria (∼ 55%), and Deltaproteobacteria (∼20%) were all common in overlying soils, while Chloroflexi (9.97%), Nitrospirae (7.19%), Gemmatimonadetes (4.61%), and Firmicutes (3.53%) were common in cave sediments. Proteobacteria in drip waters were distinctive from other habitats, specifically Gammaproteobacteria and Betaproteobacteria (**Figure 7**). Key genera identified for each karst components were *Crossiella, Euzebya*, and *Rubrobacter* for weathered rocks, *Bradyrhizobium* and *Acidothermus* for overlying soils, *Gaiella* for cave sediments, and *Sediminibacterium, Brevundimonas, Acidovorax, Hydrogenophaga, Polaromonas, Acinetobacter, Perlucidibaca*, and *Pseudomonas* for drip waters (**Figure 7**).

At the 97% similarity level, common OTUs in weathered rocks included OTU_13, which belonged to the Pseudonocardiales (Actinobacteria), and OTU_22, which was affiliated with the Solirubrobacterales (Actinobacteria) (**Figure 5**). OTU_47, which belonged to the Chthoniobacterale*s* (Verrucomicrobia), was dominant in most overlying soils (CT and MT) (**Figure 5**). OTU_54 of Moraxellaceae (Gammaproteobacteria) is the most abundant and unique in the drip water (**Figure 5**). Notably, most of the identified OTUs are putative heterotrophs according to the carbon utilization by the closest phylogenetic isolates (approximately 59.4% with relative abundances > 5‰).

### Environmental Controls on Bacterial Distribution

**FIGURE 8:**
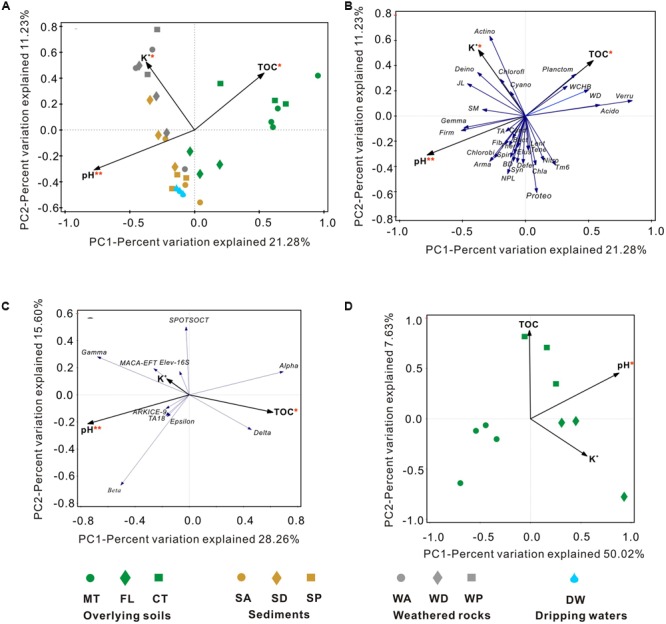
**Redundancy analysis (RDA) of 16S rRNA gene data and environmental factors at the phylum level (A,B)** and the class level in Proteobacteria **(C)** among the whole cave ecosystem, and at the phylum level only among overlying soils **(D)**. The environmental factors with *p*-values < 0.05 and *p*-values < 0.01 are marked with one red asterisk and two red asterisks, respectively. Environmental factors in gray indicate the lack of significant effects on the bacterial community structure. Sample abbreviations are described from **Figure 1**. Actino, Actinobacteria; Proteo, Proteobacteria; Acido, Acidobacteria; Chlorofl, Chloroflexi; Planctom, Planctomycetes; Bact, Bacteroidetes; Gemma, Gemmatimonadetes; Nitro, Nitrospirae; Verru, Verrucomicrobia; Firm, Firmicutes; Cand, Misc. Candidate Division groups; JL, Candidate Division JL-ETNP-Z39; Ther, Thermotogae; Cyano, Cyanobacteria; Defer, Deferribacteres; Arma, Armatimonadetes; Chla, Chlamydiae; Lent, Lentisphaerae; Deino, Deinococcus-Thermus; TA, Candidate Division TA06; WCHB, Candidate Division WCHB1-60; SM, Candidate Division SM2F11; BD, Candidate Division BD1-5; Fib, Fibrobacteres; NPL, Candidate Division NPL-UPA2; Syn, Synergistetes; Spir, Spirochaetae; Elus, Elusimicrobia; Tene, Tenericutes; WD, Candidate Division WD272.

To identify the environmental parameters that correlate with bacterial community variability across the Heshang Cave ecosystem, RDA was performed using 12 environmental factors (**Figure 8**). Among those investigated, pH (14.9% of the variability in community composition, *p*-value = 0.002), TOC (9.8%, *p*-value = 0.022) and K^+^ (7.1%, *p*-value = 0.03) were crucial factors that correlated with bacterial community compositions at the phylum level (**Figures 8A,B**). RDA analysis of proteobacterial class demonstrated 53.0% variation in total, in which pH and TOC accounted for 28.6% (**Figure 8C**).

Different phyla showed specific correlations with pH, TOC, and K^+^. In particular, decreased relative abundances of Acidobacteria (*R*^2^= 0.253, *p*-value = 0.004), Verrucomicrobia (*R*^2^= 0.5097, *p*-value < 0.0001) and Planctomycetes (*R*^2^= 0.3003, *p*-value = 0.0014) significantly correlated with an increase in pH, while higher abundances of Gemmatimonadetes and Firmicutes significantly correlated to acidic pH. Furthermore, the higher the pH, the more abundant the Gammaproteobacteria (*R*^2^= 0.167, *p* = 0.022) and Betaproteobacteria (*R*^2^= 0.213, *p* = 0.009) class representation (**Figure 8C**).

Relative abundances of Acidobacteria (*R*^2^= 0.228, *p*-value = 0.0066), Verrucomicrobia (*R*^2^= 0.1405, *p*-value = 0.0378), and Planctomycetes (*R*^2^= 0.2024, *p*-value = 0.0111) were significantly positively related to TOC content (**Figure 8B**). Bacterial communities from farmland soil, most of the cave sediment, and drip waters were positively correlated with pH and negatively correlated with K^+^ and TOC content in RDA space (**Figure 8A**). Bacterial communities in weathered rocks were plotted positively in RDA space with K^+^; Actinobacteria was the only phylum significantly correlated with K^+^ content (*R*^2^= 0.2832, *p*-value = 0.0021, **Figures 8A,B**).

**FIGURE 9:**
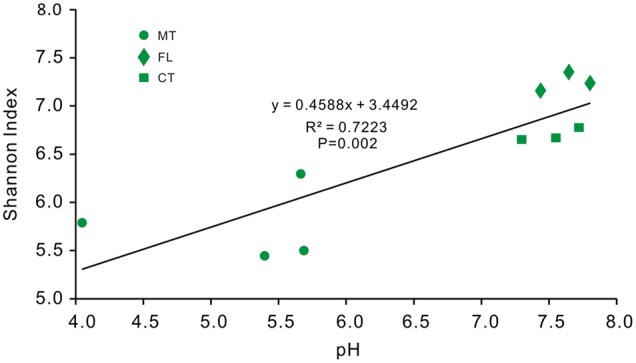
**Regression analysis between bacterial OTU diversity and pH values from overlying soil samples of Heshang Cave.** Sample colors are the same as previous figures, and abbreviations used are listed for **Figure 1**.

Lastly, the diversity of bacterial communities in surface soils was related to pH (40.3%) (**Figure 8D**), and community position in RDA space indicated a strong positive correlation between the soil bacterial diversity and pH (*R*^2^ = 0.7223, *p*-value = 0.002; **Figure 9**).

## Discussion

Soil pH is widely accepted as a critical factor impacting the compositions of soil bacterial communities (Fierer and Jackson, 2006; Nicol et al., 2008; Baker et al., 2009; Davis et al., 2009; Jenkins et al., 2009; Jones et al., 2009; Lauber et al., 2009; Chu et al., 2010; Shen et al., 2013; Liu et al., 2014). Soil pH has also been shown to strongly impact relative abundances of certain groups of bacteria in soils, including the Acidobacteria, Gammaproteobacteria, and Betaproteobacteria (Lauber et al., 2009; Shen et al., 2013).

But, karst soils and other habitats associated with a single karst system are poorly represented in studies focused on bacterial community diversity and structure (Zhou et al., 2009; Knáb et al., 2012). Therefore, our study expands this knowledge fundamentally, and the results demonstrate that pH plays a significant and important role in shifting bacterial community compositions in diverse karst habitats, and that each habitat has certain bacteria. Specifically, the most common bacteria identified from the karst soils above Heshang Cave were Acidobacteria, Verrucomicrobia, and Planctomycetes, with relative abundance of Acidobacteria and Verrucomicrobia matching those from other soils, at 20 and 23%, respectively (Janssen, 2006; Bergmann et al., 2011; Greening et al., 2015). The higher abundances of Alphaproteobacteria and Deltaproteobacteria in surface soils from the Heshang Cave ecosystem were similar to those found from other soils (Janssen, 2006; Spain et al., 2009). Heshang Cave sediments were dominated by Chloroflexi, Nitrospirae, Gemmatimonadetes, and Firmicutes, which have also been identified from cave sediments elsewhere around the word, such as in the Western Carpathians of Romania (Epure et al., 2014) and Jinjia Cave from the east Gansu Province of China (Wu et al., 2015). Actinobacteria dominated the weathered cave wall communities in Heshang Cave, and this phylum is commonly found in cave environments, such as the weathered rocks of the Buda thermal Karst System (Borsodi et al., 2012), Grotta dei Cervi in Italy (Groth et al., 2001) and other cave walls (Mulec et al., 2012a,b). Proteobacteria, specifically Gammaproteobacteria and Betaproteobacteria, are the most abundant bacteria in the drip waters, including the ones in Heshang Cave (Yun et al., 2016).

Our study also provides unique information about the relationship between environmental variability, especially pH, and bacterial community diversity and the abundance of specific groups from a single karst system. Overall, diversity indices for the soil bacterial community showed a positive correlation with pH (**Figure 9**). However, while previous studies indicate a positive correlation between Alphaproteobacteria and pH in soils (Rousk et al., 2010; Bartram et al., 2014), our results showed a negative correlation between Alphaproteobacteria and pH (*R*^2^ = 0.409, *p*-value = 0.0001). We did not observe any positive correlation between the relative abundance of Actinobacteria and pH, in contrast to previous reports (Shen et al., 2013). The discrepancies may be because the other studies were done in somewhat natural or pristine conditions related with pH (Lauber et al., 2009; Shen et al., 2013). It is likely that surface soils near Heshang Cave have already become acidified because of increased acid rainfall in the central China (**Figure 1A**). As such, the bacterial diversity that we uncovered may have already adapted to decreasing pH. But, because we evaluated the community compositional changes at low taxonomic resolution, it is possible that higher taxonomic resolution (i.e., at the order- and familial-levels) may reveal more detailed effects of pH change on community composition. Future research should compare results from pristine soils with those known to be impacted by acidification, as well as focus on diversity changes at higher taxonomic resolution. For instance, changes in pH and lower NH_3_ in a soil environment (Xu and Gao, 2011) have been linked to shifts from ammonia-oxidizing bacteria to archaea (Zhao et al., 2016). Recently, soil acidification due to increasing acid rainfall in China was linked to a decrease in the microbial abundance (Wu et al., 2006; Xu et al., 2015), as well as the diversity (Zhalnina et al., 2015), which may result in a decrease in microbial functional diversity because functional gene diversity is significantly and positively correlated with taxonomic diversity (Fierer et al., 2013).

Moreover, pH also plays a significant role in controlling the rates of microbial decomposition of organic matter (Wakelin et al., 2008). Although most cave systems are predominately oligotrophic habitats (Barton and Jurado, 2007), heterotrophs, which fed on organic matter, account for ≥75% of microbial communities in many caves (Mikell et al., 1996). Our results showed that the closest relatives associated with Planctomycetes (Fuerst, 1995), Verrucomicrobia (Hedlund et al., 1997), and Acidobacteria (Ward et al., 2009) were either chemoorganotrophs or aerobic heterotrophs. These putative metabolisms were highly dependent on organic matter, which explains the relationship between TOC with bacterial community composition in both soil and cave samples, specifically the relative abundances of Actinobacteria, Verrucomicrobia, Gammaproteobacteria, and Deltaproteobacteria (**Figures 8B,C**). These results are also confirmed by previous studies (Liu et al., 2014). Because the decrease of pH from 8.5 to 7.4 dramatically increases the decomposing rates of organic matter (Leahy and Colwell, 1990) by heterotrophs, the alkaline status of karst soils and related habitats would maintain a lower decomposition rate of organic matter. However, soil acidification and penetration of acidic fluids to greater depths within a karst system due to the increase in the amount of both precipitation (Pu et al., 2011) and acid rainfall can remarkably enhance microbial decomposition of organic matter in karst areas and result in an increase of CO_2_ release in the future, which would in turn likely cause a linked effect between pH and TOC on microbial activities in karst ecosystems.

Besides pH and TOC, potassium content also significantly correlated with bacterial communities in the Heshang Cave ecosystem. Potassium, which can affect the functions of transporters through controlling the cytosolic ionic strength (Saxena et al., 2015), can also affect microbial community diversity and composition (Mendes et al., 2014; Pereira et al., 2014; Stroobants et al., 2014; Archer et al., 2015; Li et al., 2015; Saxena et al., 2015). K^+^ and pH correlated to specific taxonomic groups, such as *Bacillus* and *Pseudomonas*, in the cave sediments and drip waters, respectively (**Figure 7**), and these bacteria may play a dominant role in releasing potassium into the environment during pH changes within the karst system (Hu et al., 2006; Mendes et al., 2014).

In summary, the results from this study indicate that soil acidification due to the increase in acid rainfall and enhanced monsoonal precipitation in central China is expected to impact the bacterial communities throughout the entire karst system, from surface soils to percolating epikarst waters, to cave sediments and rocks. The bacterial communities that are mostly affected by acidification will be those in areas of the karst where carbonate rock dissolution and pH buffering will diminish through time, such as from inside the cave. The impact is attributed to the penetration of acidic solutions into the deep karst system, as well as increased rates of decomposing organic matter decomposition in karst soils and shallow epikarst under acidic conditions. This will further decrease the TOC that may otherwise enter the subsurface. Higher rates of organic matter decomposition due to acidification will also increase the release of CO_2_ into the soils and epikarst waters, which will lead to enhanced carbonate rock dissolution and then allow additional acidic fluids to penetrate to greater depths in the subsurface karst. Additional research should target understanding the rates of organic matter decomposition by specific bacterial groups that are vulnerable to acidification in the alkaline habitats of the central China karst.

## Author Contributions

YY completed the sample collection, DNA extraction, data analysis, and prepared the manuscript draft. HW designed the experiment, analyzed the data, and wrote the manuscript. BM, XX, and AE assisted with the partial data analysis. JZ, XQ, and YD helped with sample collection. AE helped with polishing the text and improving the structure and logic of the manuscript.

## Conflict of Interest Statement

The authors declare that the research was conducted in the absence of any commercial or financial relationships that could be construed as a potential conflict of interest.

## Footnotes

1 http://www.ncbi.nlm.nih.gov/sra/SRP068087

2 http://ccb.jhu.edu/software/FLASH/

3 http://greengenes.secondgenome.com/

4 http://blast.ncbi.nlm.nih.gov/Blast.cgi

5 http://www.mothur.org/wiki/Simpson

6 http://www.mothur.org/wiki/shannon

7 https://cran.r-project.org/bin/windows/base/old/3.2.0/

8 http://spss.software.informer.com/17.0/
